# Timing of Health Service Use Among Truck Drivers After a Work-Related Injury or Illness

**DOI:** 10.1007/s10926-021-10001-y

**Published:** 2021-09-08

**Authors:** Ting Xia, Alex Collie, Sharon Newnam, Dan I. Lubman, Ross Iles

**Affiliations:** 1 Insurance Work and Health Group, Faculty of Medicine, Nursing and Health Sciences, Monash University, 553 St Kilda Rd, St Kilda, VIC 3004 Australia; 2 Monash Accident Research Centre, Monash University, Building 70, Monash University Clayton Campus, Clayton, VIC 3800 Australia; 3 Turning Point, 110 Church St, Richmond, VIC 3121 Australia; 4 Eastern Health Clinical School, Monash University, Level 2, 5 Arnold Street, Boxhill, VIC 3128 Australia

**Keywords:** Work-related injury and illness, Health service use, Truck driver, Occupational injury recovery

## Abstract

*Purposes* Timely delivery of treatment and rehabilitation is generally acknowledged to support injury recovery. This study aimed to describe the timing of health service use by injured truck drivers with work-related injury and to explore the association between demographic and injury factors and the duration of health service use. *Methods* Retrospective cohort study of injured truck drivers with accepted workers’ compensation claims in the state of Victoria, Australia. Descriptive analyses examined the percentage of injured truck drivers using health services by service type. Logistic regression model examined predictors of any service use versus no service use, and predictors of extended service use (≥ 52 weeks) versus short-term use. *Results* The timing of health service use by injured truck drivers with accepted workers’ compensation claims varies substantially by service type. General practitioner, specialist physician, and physical therapy service use peaks within the 14 weeks after compensation claim lodgement, whilst the majority of mental health services were accessed in the persistent phase beyond 14 weeks after claim lodgement. Older age, being employed by small companies, and claiming compensation for mental health conditions were associated with greater duration of health service use. *Conclusions* Injured truck drivers access a wide range of health services during the recovery and return to work process. Delivery of mental health services is delayed, including for those making mental health compensation claims. Health service planning should take into account worker and employer characteristics in addition to injury type.

## Introduction

Across many jurisdictions globally, funding healthcare is one of the main mechanisms by which workers’ compensation schemes assist injured workers in their recovery. As part of the workers’ compensation process, healthcare workers provide a vital role in examining injured worker’s conditions, determining the work-relatedness of injury, providing treatment and rehabilitation [1]. Thus, compensation schemes are critical in supporting not only injured workers and the workplace, but healthcare providers play a key role in facilitating an injured worker’s return to work.

Prior research suggests that the administrative complexity of workers’ compensation systems [2] and lack of understanding of insurance processes among healthcare providers can impact the efficiency of healthcare provision, and this can in turn contribute to poorer care [3]. One challenge impeding successful outcomes is the delivery of timely healthcare. There is much research to suggest that timely healthcare delivery supports injury recovery, and thus contributes to improving workability [4]. For example, providing appropriate treatment for mental health issues at an early stage has been found to be positively associated with quality and speed of recovery [5]. Conversely, delays in providing psychological treatment to workers with mental health conditions have been linked to poorer outcomes including social and cognitive decline [6]. Increased time from injury to treatment was also found to be associated with poorer self-reported quality of life [7]. However, a longer duration of healthcare may not add additional benefit in preventing work-disability recurrence or reducing work-disability duration [8].

A second challenge impeding successful outcomes relates to accessing healthcare. Previous research has demonstrated that both intensity and patterns of service use are influenced by demographic and social factors beyond the nature of the injury or disease the worker is experiencing [9]. Unique clinical challenges in some injured worker cohorts have been reported as major barriers to effective healthcare delivery [10]. For example, accessing appropriate healthcare is challenging for workers who regularly experience long working hours, time pressure, irregular shifts, and financial stress in their jobs.

Truck driving is one example of an occupation that may inhibit access to timely healthcare. In addition to the challenges related to their working environment (e.g. long working hours and work away from home), truck drivers are also likely to live in rural and regional locations which further limits access to healthcare [11]. This issue warrants investigation given that in Australia, truck driving is the most common occupation for males, with 1 in 33 men being a driver [12]. Truck driving is also a predominantly male occupation with a rapidly aging workforce, thereby there is an increased need to maintain the health of the current workforce [13]. Workers’ compensation data shows that truck drivers have higher rates of work-related injury and illness when compared to other industries and occupations [14]. Recent studies have highlighted an increasing prevalence of musculoskeletal injuries, mental health conditions, and other chronic conditions in this population [15, 16]. However, there is very little published evidence regarding health service use (HSU) in this group.

Previous research has identified that several factors influence the timing of HSU, including the nature and severity of injury [17], whether the healthcare delivery system is guided by a clinical framework [18], as well as policy settings such as rules regarding maximum claim processing time [2]. Given truck drivers’ unique occupational characteristics, understanding the timing and nature of HSU among the truck driving workforce is an important step in developing effective approaches to occupational health and safety and injury management. The first aim of this retrospective cohort study was to describe the timing of HSU by truck drivers with accepted workers’ compensation claims. The second aim was to examine the association between demographic and injury factors and the duration of HSU.

## Method

### Study Population and Setting

The state of Victoria, Australia operates a cause-based workers’ compensation system, in which workers may be eligible to receive income benefits, healthcare and other services following a workplace injury or disease, if that injury or disease can be attributed to the circumstances of work. Under the Victorian workers’ compensation scheme, when liability has been accepted on a claim an employer is liable for the first 10 days of incapacity and the first $744 (indexed annually) of medical and like expenses. Workers may be eligible to receive income replacement for a period of up to 130 weeks, and in most cases, a worker will be eligible to access funding for health care related to their compensable injury for up to 52 weeks from cessation of income support. However, compensation for medical and like services may continue beyond the time limits under some circumstances (e.g. the worker has returned to work but could not remain at work if the service is not provided or surgery is required) [19].

The Victorian scheme covers approximately 85% of all workers in the state [20].The other 15% are either sole traders (who do not need to register for workers’ compensation in Victoria), employees of self-insurers, or workers covered under the Comcare workers’ compensation scheme (mainly federal government employees).

### Data and Outcomes

This study used data from a workers’ compensation dataset provided by the regulator of the state workers’ compensation system. Data extracted from the compensation dataset consisted of ‘accepted’ claims (i.e., those have been accepted for payment) lodged by truck drivers with a date of lodgement between 1/07/2004 and 30/06/2014, and daily observation records of individual episodes of HSU in the 12-week period prior to claim lodgement and up to 130 weeks post claim lodgement. Date of lodgement was defined as the date that the workers’ compensation insurer received and date-stamped the workers’ compensation claim form. The date of lodgement was chosen for data extraction as there could be possible a gap between injury date and claim lodgement date in some claims. For instance, the time lag between lodgement date and injury date ranged from 0 to 3863 days for musculoskeletal injury (MSK), with a median time lag of 23 days (interquartile range (IQR): 13–53 days). For mental health claims, the time lag between lodgement date and injury date ranged from 0 to 3422 days, with a median time lag of 26 days (IQR: 14–80 days). Therefore, we only use reliable health service data for the period close to lodgement and after. Truck drivers were identified using the Australian and New Zealand Standard Classification of Occupations (ANZSCO) codes (3-digit code 733) [21]. Analysis of HSU records focused on those episodes where there was a worker–provider interaction and excluded services with descriptions that indicated there was no direct engagement between the worker and the healthcare provider (e.g., report writing and review forms consultation, work site visit and audiovisual viewing by GPs).

In this study, we examined seven categories of service use, primarily grouped by provider type based on Australian Medicare Benefits Schedule (MBS) categories: general practitioner (GP), specialist physician, mental health, surgery, return to work (RTW) services, physical therapy and independent medical examination (IME). The extracted health service payment data were limited to those episodes where there was a patient‐provider interaction and excluded services with descriptions that suggested there was no direct engagement between the worker and the healthcare provider (eg, psychology notification and review forms consultation, work site visit, and audiovisual viewing by GPs). Therefore, ambulance services, nursing, diagnostic imaging and pathology services were also excluded from the analysis. Moreover, using MBS codes limited to identify patient-doctor interactions occurred within the hospital in the payment data.

Therefore, only hospital admissions resulting in surgery were included into the analysis.

The description of services included for each service type category is presented in Table 1.

**Table 1:** Service type categories and description of services included

Service type category	Service items included
General practitioner (GP)	All professional attendances by a general practitioner excluding GP mental health treatment plans
Specialist physician	All professional attendances by a specialist physician excluding independent medical examinations, psychiatry and surgery
Mental Health	All professional attendances by a psychiatrist or a psychologist, and Psychological Therapy Services, GP Mental Health Treatment Items, and Allied Mental Health but excluding medical assessment
Surgery	An episode of Surgery that resulted in hospital admission (at least one day of hospital stay) OR episodes of surgery in which there were ‘theatre’ or 'accommodation' use recorded
Return to Work	Counselling, assessments and consultations from (a) Occupational rehabilitation; (b) Rehabilitation services; (c) Internal rehabilitation providers; (d) External rehab providers
Physical therapy	Professional attendances by a physiotherapist, chiropractor, osteopath as well as remedial massage, but excluding medical assessment
Independent medical examinations (IME)	Independent medical examinations conducted by a medical doctor or allied health practitioner

The analysis included episodes of HSU that occurred in the period two months (13 weeks) prior to claim lodgement and up to 2.5 years (130 weeks) post claim lodgement, which is the period of potential income replacement. These time periods were chosen as the insurer may ‘back-pay’ for health services rendered for work-related injury/illness that occurred prior to the date of claim lodgement when they accept the compensation claim. In this study, extended service use was defined as any service use occurring more than one year after claim lodgement.

### Factors Associated with Duration of Health Service Use

The factors associated with the duration of HSU were chosen based on prior evidence in the research literature [22, 23] and data available in the workers’ compensation dataset. Demographic and injury details were derived from the claims dataset including sex, age, socioeconomic status, employer size, type of injury, mechanism of injury and type of claim. Age refers to drivers’ age at the time of injury/illness. Age was converted to a categorical variable by 10-year grouping. (≤ 24, 25–34, 35–44, 45–54, 55–64, 65 + years old). Socioeconomic status was defined by the Index of Relative Socioeconomic Advantage and Disadvantage (IRSAD) [24] from 1 (the most disadvantaged area) to 5 (the least disadvantaged area). Employer size was classified as small, medium, large or government based on the employer's remuneration in 2010/11 deflated to 2005/06 dollars: < $1 million = small, $1 to $20 million = medium, and > $20 million = large. Types of work-related injury and illness were categorized using a modified version of the type of occurrence classification system (TOOCS) version 3[25] and we focused on six major categories of injury types, including fractures, musculoskeletal injury (MSK), neurological injury, mental health injury, other traumatic injury, and other diseases. Type of claim was defined to indicate whether the worker received payments for income loss (time loss claim) or medical expenses only (medical only claim).

### Analysis

An initial series of descriptive analyses were performed to examine the percentage of injured truck drivers using health services by service type. The median number of episodes per injured worker was also described for each service category. Second, the aggregated weekly total episodes of service use for each service category over the entire follow-up time period were presented as a density plot. The association between demographic and injury factors and service use was tested in two models: a logistic regression model relating predictors of any service use versus no service use (Model 1), and a logistic regression model relating predictors of extended service use (≥ 1 year) versus short-term use (Model 2). For Model 2, only truck drivers with at least one studied service use episode were included. Both models were adjusted for all noted demographic and injury characteristics.

## Results

In total, we included 13,371 accepted work-related injury and illness claims from truck drivers, and 71% had at least one included service use record in the claim dataset. As shown in Table 2, GP, physical therapy and specialist physician were the most common health services used by injured truck drivers. Around 55% of injured truck drivers attended a GP service, and about 40% had a specialist physician consultation or physical therapy visit. Nearly one third of injured truck drivers underwent major surgery, while mental health services were used by only 6.3% of injured truck drivers. The median number of visits among those who received physical therapy was 16 (IQR: 6 to 39) over the 130 weeks post injury. Although mental health service was the least common health service accessed by truck drivers, there was a longer median duration of utilisation when mental health services were accessed, second only to physical therapy.

**Table 2:** Percentage, median and interquartile range (IQR) of health service used by injured truck drivers

Health service type	% of accepted claims	Median (IQR) service use where at least one service was used
GP	55.43%	6 (3–18)
Specialists	39.87%	3 (1–6)
Mental health	6.27%	9 (4–23)
Surgery operation (major)	26.37%	1 (1–1)
RTW service	24.02%	2 (1–4)
Physical therapy	40.24%	16 (6–39)
IME	10.77%	1 (1–1)

**Fig. 1:**
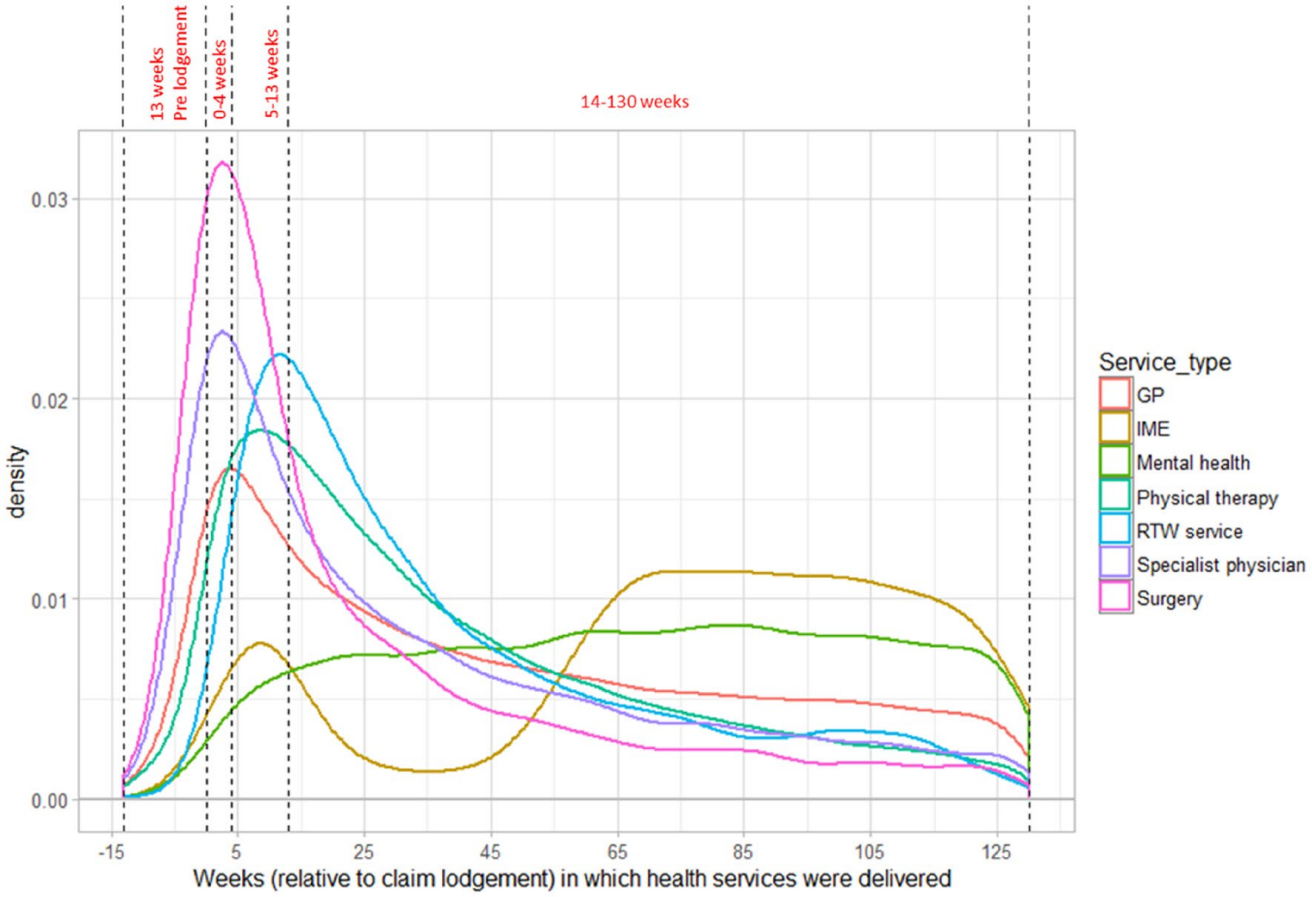
Density plot of health service use by service type

The density plot (Fig. 1) represents the distribution of each type of HSU for truck drivers from 13 weeks before claim lodgement to 130 weeks after claim lodgement. Overall, GP service use peaked between 2 weeks before and 10 weeks after claim lodgement and then decreases gradually. The use of specialist physician services peaked between one week before and after claim lodgement. Physical therapy service use peaked between 5 and 15 weeks after claim lodgement before declining steadily up to the conclusion of the 130 week follow up period. The use of mental health services shows a different pattern compared to other service types. We found less use of mental health services within the first 5 weeks after lodgement, increasing sharply and then remaining stable up to 130 weeks following claim lodgement. We also observed two peaks in the pattern of independent medical examination. The first peak occurred around 5 to10 weeks after claim lodgement and the second peak started around 55 weeks following lodgement.

**Fig. 2:**
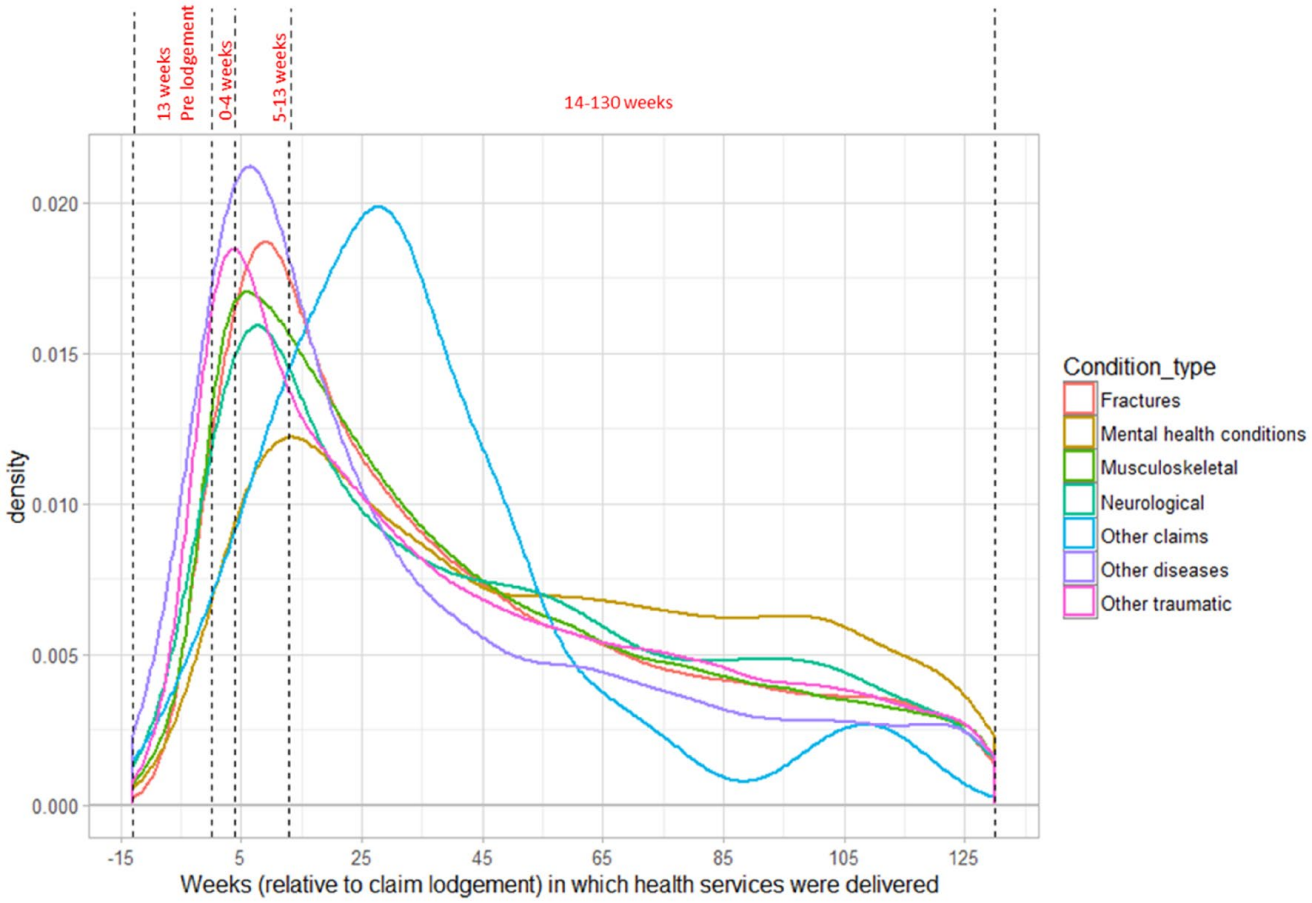
Density plot of health service use by injury type category

Figure 2 shows the density of HSU for each type of injury in truck drivers from 13 weeks before claim lodgement to 130 weeks after claim lodgement. The peak density occurred before 13 weeks for all types of injury except mental health conditions and other claims. Around 30% of the HSU for fractures, musculoskeletal and neurological injuries occurred within the first 4 weeks after claim lodgement. In contrast, the majority of HSU for mental health conditions (82%) was observed to be in the persistent phase beyond 14 weeks post lodgement.

**Fig. 3:**
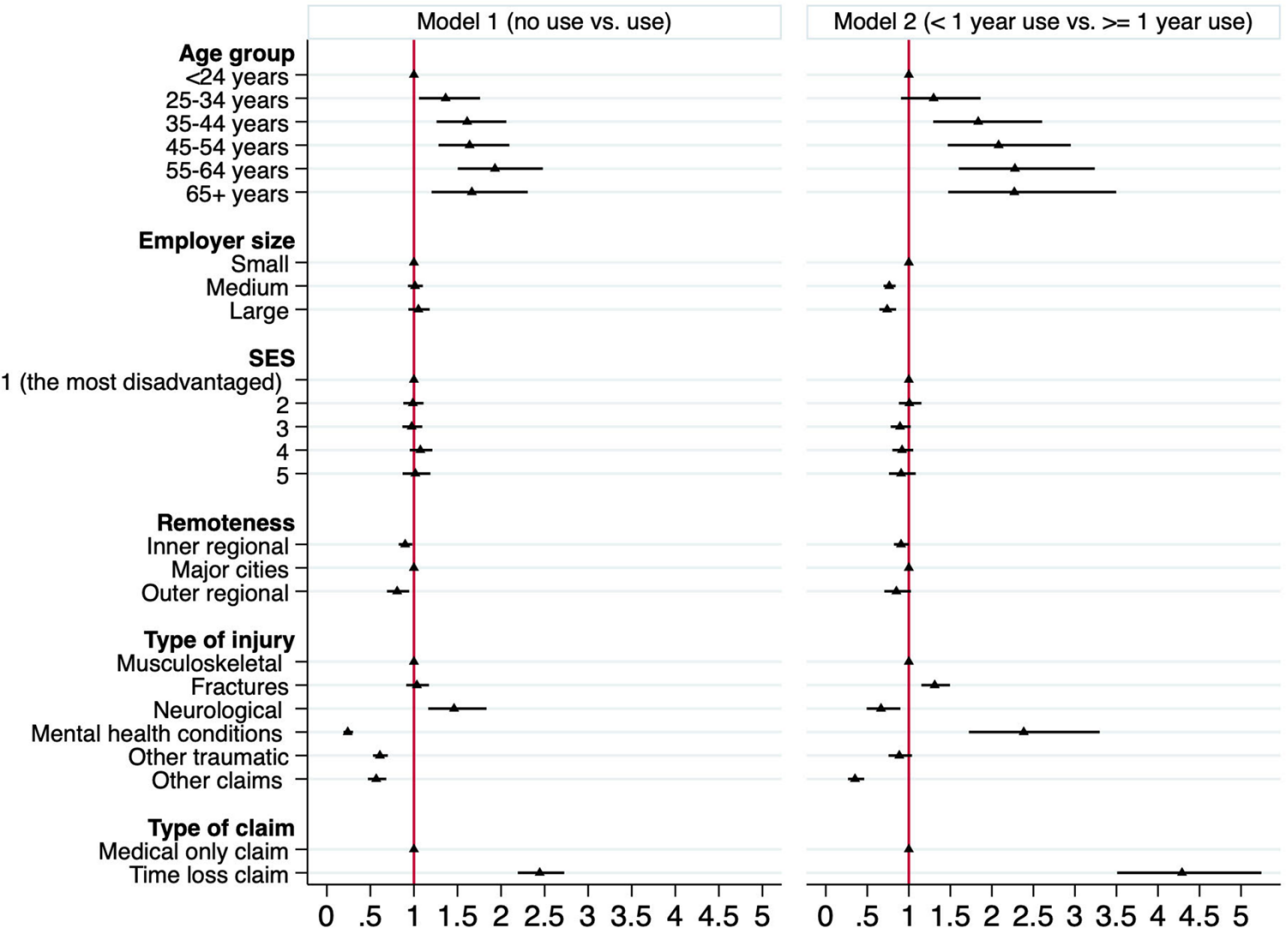
Odds ratios of the factors of duration of health service use. Note: a confidence interval that does not cross the ‘1’ line means results are statistically significant (p < 0.05); SES = social economic status

Figure 3 plotted the odds ratios of the predictive factors of any service use versus no service use (Model 1), and predictive factors of extended service use versus short-term use (Model 2). The confidence interval that does not cross the ‘1’ line means the results are statistically significant. Younger truck drivers, particularly those under 24, were most likely to not use health services post injury. Truck drivers aged 55–64 years had the greatest odds for health service use (OR 1.93, 95% CI 1.50–2.48) than those under 24 years. Compared with drivers with musculoskeletal injury claims, those with mental health claims had 75% decreased odds of using any of included health services (OR 0.24, 95%CI 0.19–0.30). Employer size and SES were not significant predictors of service use, whilst drivers who lived in inner regional areas and outer regional areas were associated with decreased odds of using any of included health services compared to those lived in major cities (OR 0.90, 95% CI 0.82–0.98 & OR 0.81, 95% CI 0.69–0.95). Time loss claims had a greater odds for health service use (OR 2.44, 95%CI 2.19–2.73) than medical only claims.

As shown in Model 2, among those who used health service post injury, extended service use was associated with increasing age, with a maximum for those aged 55–64 years old (OR 2.28, 95%CI 1.60–3.24) followed by those aged in 65 years or more (OR 2.27, 95% CI 1.47–3.50). Extended service use was associated with truck drivers who were employed by medium or large companies (OR 0.76, 95% CI 0.69–0.84 & OR 0.74, 95% CI 0.65–0.85). Making a claim for a mental health condition was a significant predictor of extended service use with drivers making such claims having a 2.38 times greater odds (95%CI 1.72–3.30) compared to those making musculoskeletal injury claims. Drivers making neurological claims and other claims had the lowest odds of having extended service use (OR 0.66, 95% CI 0.49–0.90 & OR 0.35, 95% CI 0.26–0.46). Compared with medical only claims, time loss claims had 4.29 times greater odds (95% CI 3.51–5.24) of extended health service use. Remoteness was not found to be associated with extended service use in the Model 2.

## Discussion

The findings from this study provide new insight into the timing of HSU following work-related injury and illness among truck drivers. In general, physical therapy and GP services were the most commonly used health services while mental health, independent medical examination and return to work services were the least commonly used. The duration of HSU was associated with age, employer size and type of injury.

We observed that the timing of HSU in the workers’ compensation system varies substantially by service type. General practice, specialist physician and surgical services peaked within the acute period post-injury. In particular, surgery often occurred before or around the date of claim lodgement and GP and specialist physician services commonly occurred in the few weeks after claim lodgement. These findings may reflect both the nature of injuries incurred by truck drivers and workers’ compensation policy factors. In Australia, GPs see over 90% of injured workers[26], and a certificate of capacity to the employer from the GP is required to lodge a claim [10]. It is not surprising then that there was greater GP appointment density in the early stages after injury. Truck drivers are also at higher risk of acute traumatic injury through road traffic crashes [11, 27]. When a surgery is required, a request will usually need to be completed and the surgery approved prior to any procedure and/or hospitalization. However, this policy is not applicable in the case of an emergency. This could be explained by the multiple uses of IMEs in compensation systems. Medical examinations in the early period post claim may be requested by the workers’ compensation insurer where there is a question about the medical diagnosis and also to determine the prognosis, or likely duration of disability [28]. Therefore, the first peak of using IME may be about determining the appropriateness and effectiveness of health services in claims that exceed three months duration. Examinations later in the rehabilitation period relate to workers with slower recovery, and thus the second peak could be explained as requests to provide opinions on the drivers’ current work capacity and ongoing rehabilitation needs.

The large proportion of truck drivers who accessed physical therapy align with the findings of our previous work showing that musculoskeletal injury is the most common work-related injury for Australian truck drivers [11]. The use of physical therapy peaked in the sub-acute period between 5 and 13 weeks after claim lodgement and the median number of physical therapy sessions was higher than other services. The high volume and persistent use of physical therapy may be explained by the high risk of sustaining chronic musculoskeletal conditions such as low back pain among this occupational group [29, 30].. However, a recent systematic review suggested that physical therapy treatment choices for musculoskeletal conditions are often not based on evidence-based guidance [31]. They reported there was extensive use of not-recommended treatments and treatments without recommendations [31]. Our study highlights the significance of musculoskeletal injuries in Australian truck drivers, however, whether they received best-practice treatment requires further study.

Our findings highlight that the pattern of mental health service use post-injury is very different to that of other health services. We observed that the use of mental health services peaked in the period beyond 14 weeks from claim lodgement, and injured truck drivers with mental health conditions could have persistent healthcare needs that extend beyond 2.5 years. Our findings of relatively low use of mental health services in the early phase of injury suggest there may be missed opportunities for early intervention in the initial stages of a mental illness. While it is possible that delivery of mental health services later in a claim may be appropriate as ongoing adjustment to injury and claim processes can lead to the development of secondary mental health concerns [32, 33], it has been shown that just 27% of claimants experiencing psychological distress alongside making a claim for a musculoskeletal condition report accessing mental health services[17]. Furthermore, mental health issues can develop later due to delayed injury impacts. For instance, a recent study suggested that stressful interactions with claims case managers can lead to a serious mental illness at late stage following a workplace injury or illness [34]. Previous studies have shown that early intervention in mental illness can have a significant positive impact on a patient’s prognosis, whereas treatment delay is independently associated with poor outcomes [35]. This late initiation of mental health services may be attributable, in part, to the time taken to refer to secondary and usually more specialised services from the primary health care setting. For instance, a previous study suggested that claim processing times were consistently longer for claims involving neurological and mental health conditions [3]. In addition, there can be a delay in mental health condition recognition for this occupation since the treatment team may focus on managing acute injury thereby seeing distress as an appropriate reaction to injury. However, truck drivers experience a host of occupational stressors that are embedded within the transportation environment that put drivers at risk for depression and anxiety [36], which require a longer period of time to recover or treat. Therefore, for drivers with mental health claims, some level of support (e.g. GP support) in the first instance provided by the compensation scheme may be needed.

Another noteworthy finding was that while there wasn’t a high prevalence of mental health service use, those drivers who did claim for mental health conditions tended to require long term support from the compensation system. It has been previously documented that truck drivers are at high risk of poor mental health, and truck drivers with work-related mental health conditions spend significantly longer duration of time off work compared to drivers with musculoskeletal conditions [11]. However, they face difficulties accessing appropriate health care because of long working hours, not being at home and unpredictable schedules. Therefore, more timely and targeted referrals to specialist services, including mental health services, might be the key to supporting driver recovery once injuries occur.

The regression model revealed that older age was associated with greater volume and duration of HSU. Truck drivers aged 55–64 years had the greatest risk of extended service use. This may partially be due to a higher prevalence of work-related chronic conditions among older truck drivers [37]. Previous studies reported the largest proportion of work-related injuries occurred among truck drivers between 40 and 60 years of age [11]. Past studies also suggest that musculoskeletal injuries are most common among long-haul truck drivers with an average of 15 years of work experience [15]. Therefore, older more experienced drivers may be more likely to experience chronic conditions which require long-term treatment [37]. A sustainable approach to addressing this issue could be to target younger drivers in an effort to improve the physical and psychological health and wellbeing of drivers throughout their careers, rather than solely treating illness and disease at older ages. This could be achieved through targeted communication with healthcare providers on the risks associated with a driving career educating drivers and employers on the benefits of healthy living, as well as developing evidence-based health interventions.

We also observed that working for larger employers meant HSU was more likely to be shorter term. One explanation for this could be the importance of employer support for a successful return to work after injury. Compared with small employers, larger transport companies may have greater resources to devote to return to work programs, including employing skilled return to work coordinators and being able to incorporate a variety of return to work plans [38]. Drivers employed by large companies might also have better ability to access health services in a timely fashion because of greater flexibility in working time rearrangement, which could support faster recovery [39], whereas those working for smaller employers have less ability to take time out for seeking treatment/ health services. Large companies are also more likely to offer more support programs for employee bonding. These may contribute to faster recovery and early return to work outcomes, resulting in less healthcare demand. Our findings of greater odds of extended service use in workers with time loss claims demonstrates that workers with more severe or more complex injuries resulting in time away from work are more likely to have longer-term healthcare needs. However, workers may be certified to fit to return to work even if they have not yet fully recovered from a work-related injury that still required continued treatment. This means the number of days away from work may not indicate the severity or extent of harm suffered by workers.

### Strengths and Limitations

To our knowledge, this is the first study to examine the timing of health services accessed by truck drivers within a workers’ compensation system environment. The database used for this study has population coverage of workplaces, and the longitudinal nature of the data provides the opportunity to track individuals’ detailed service level information per use basis. The data allows examination of the type and duration of healthcare service use across multiple healthcare providers and service types over time. In addition, the service data was collected directly from the health providers which obviates recall biases apparent in survey based studies of HSU. However, there are some limitations in the use of this dataset. First, workers’ compensation in the state of Victoria does not cover self-employed workers. It is estimated that anywhere up to 14% of drivers are effectively self-employed, and as a result our sample does not capture all cases of work-related injury and illness in truck drivers. Second, the database does not capture HSU beyond that funded by the workers’ compensation system, for example for pre-existing conditions. Third, although there is limited evidence on cost-shifting in Australian workers’ compensation jurisdictions, there is substantial healthcare funded by other means for non-compensable injury/illness while people are on claim. Therefore, within this database, it is possible that not all episodes of health services (e.g. acute hospital admission) are captured as payments may be covered by the Australian universal health system or private health insurers in some instances. In addition, HUS presented here can be underestimated since some service use payments may have been captured in the employer-paid medical excess, and this applies to short-term HSU in particular. Using claim lodegment date for data extraction may also cause underestimation of service use especially those occurred in the acute injury period. Fourth, the compensation data only recorded primary work-related conditions, therefore we were not able to analyses the impact of comorbid conditions or other conditions developing secondary to the primary condition. Finally, variables that might influence the duration of HSU, such as injury severity, pre-injury or co-morbid health conditions, are absent from the database and thus could not be incorporated into the analysis.

## Conclusion

This study is the first to explore the timing and factors associated with HSU for work-related injury and illness in Australian truck drivers and has provided valuable evidence to better prioritise the allocation of healthcare resources to optimise the health and wellbeing of this growing workforce. Results demonstrate that the timing of health service use varies by service and injury type. Our findings of relatively low use of mental health services in the early phases of injury are noteworthy, and potentially reflects a missed opportunity for early intervention in the initial stages of mental illness. Our study also suggests that injury management practices for the trucking industry should prioritise medical resources to older drivers, treatment for chronic musculoskeletal conditions and timely referrals for mental health injury.

## Declarations

### Availability of Data and Material

Data used in this paper are not available for distribution by the authors.
